# Id4 Marks Spermatogonial Stem Cells in the Mouse Testis

**DOI:** 10.1038/srep17594

**Published:** 2015-12-01

**Authors:** Feng Sun, Qing Xu, Danfeng Zhao, Charlie Degui Chen

**Affiliations:** 1 State Key Laboratory of Molecular Biology, Institute of Biochemistry and Cell Biology, Shanghai Institutes for Biological Sciences, Chinese Academy of Sciences, Shanghai 200031, China.; 2 Shanghai Key laboratory of Molecular Andrology, Institute of Biochemistry and Cell Biology, Shanghai Institutes for Biological Sciences, Chinese Academy of Sciences, Shanghai 200031, China.

## Abstract

Mammalian spermatogenesis is a classic adult stems cell–dependent process, supported by the self-renewal and differentiation of spermatogonial stem cells (SSCs). However, the identification of SSCs and elucidation of their behaviors in undisturbed testis has long been a big challenge. Here, we generated a knock-in mouse model, Id4-2A-CreERT2-2A-tdTomato, which allowed us to mark Id4-expressing (Id4^+^) cells at different time points *in situ* and track their behaviors across distinct developmental stages during steady-state and regenerating spermatogenesis. We found that Id4^+^ cells continue to produce spermatogonia, spermatocytes and sperm in mouse testis, showing they are capable of self-renewal and have differentiation potential. Consistent with these findings, ablation of Id4^+^ cells in mice results in a loss of spermatogenesis. Furthermore, developmental fate mapping reveals that Id4^+^ SSCs originate from neonate Id4^+^ gonocytes. Therefore, our results indicate that Id4 marks spermatogonial stem cells in the mouse testis.

Stem cells are defined universally by their ability to maintain and regenerate the anatomy and function of an adult tissue^1^. Mammalian spermatogenesis is a classic adult stem cell–dependent process, supported by self-renewal and differentiation of spermatogonial stem cells(SSCs)^2^. SSCs are stem cells of the male germ line that support the production of numerous sperm on a daily basis throughout the adult life of a male. Their ability for maintenance of steady-state spermatogenesis and spermatogenesis regeneration after damage is the only unequivocal parameter that defines SSCs^3^. In the mouse testes, normal spermatogenesis is maintained by a small subset of undifferentiated spermatogonial cells that self-renew and have actual stemness; in regenerating tissue, a second subpopulation that normally differentiates is able to self-renew and therefore probably has stemness potential^4^,^5^,^6^,^7^,^8^,^9^. However, it has long been a big challenge to identify SSCs and elucidate their behaviors in undisturbed testis.

Spermatogonial transplantation is a gold standard and one of the reliable assays to study SSC activity^10^,^11^. The transplantation technique allows only those cells that continuously self-renew and differentiate to regenerate complete spermatogenesis in the recipient. An advantage of this approach is that it determines the absolute number of functional SSCs^12^,^13^,^14^,^15^. A weakness of the transplantation approach is that it focuses on stemness potential and can’t measure actual stemness of cells in undisturbed testis. In recent years, *in vivo* lineage tracing has evolved into a powerful technique for experimentally testing the actual stemness of cells in their physiological context^16^,^17^,^18^,^19^, which provides an effective tool to study SSCs in the steady state^5^,^6^,^7^,^8^,^9^.

The inhibitor of differentiation (Id) family of helix-loop-helix proteins is a group of evolutionarily conserved molecules that play important regulatory roles in organisms ranging from Drosophila to humans. Expression of Id proteins is typically high in embryonic and adult stem/progenitor cells but levels decrease as the cells differentiate^20^. Id proteins regulate stem-cell homeostasis and fate commitment in various cell types, including neuronal^21^,^22^,^23^,^24^, hematopoietic^25^,^26^, mammary^27^, and embryonic cells^28^. For example, Id4 is required for neural stem cell proliferation and differentiation^29^. ​​Id4 is also a key regulator of mammary stem cell self-renewal and marks a subset of mammary stem cells and basal-like breast cancers with a putative mammary basal cell origin^27^. In the mouse testes, expression of Id4 is selective for A_single_ (singly isolated cells) cells within the spermatogonial population and plays an important role in the regulation of SSC self-renewal^30^. Moreover, recent study has demonstrated that Id4-expressing (Id4^+^) cells have regenerative capacity in SSC transplantation experiments^31^. However, transplantation assays do not reveal *in vivo* stem-cell behavior in its physiological context. It is not clear whether the actual stemness could have been observed if the cell had been studied in its endogenous environment, before isolation and transplantation.

Lineage tracing measures the actual stemness of cells in their physiological context^18^. To identify SSCs and elucidate their behaviors in undisturbed testis, we used an *in vivo* lineage tracing approach to study the contribution of Id4^+^ cells to spermatogenesis and differentiation in the undisturbed testis. For this purpose, we have generated a knock-in mouse model, Id4-2A-CreERT2-2A-tdTomato, which allows us to mark these cells at different time points *in situ* and to track their behavior across distinct developmental stages during steady-state and regenerating spermatogenesis. Here, our study demonstrated that Id4^+^ cells continuously give rise to spermatogonia, spermatocytes, and sperm in undisturbed testis and during regenerating spermatogenesis, documenting their ability to self-renew and their differentiation potential. Consistent with these findings, ablation of Id4^+^ cells in mice resulted in a disruption of spermatogenesis. Furthermore, lineage-tracing studies with neonatal mice revealed that Id4^+^ SSCs are derived from neonate Id4^+^ gonocytes.

## Results

### Generation and Identification of the Id4-CreERT2-tdTomato Knock-in Mouse

**Figure 1:**
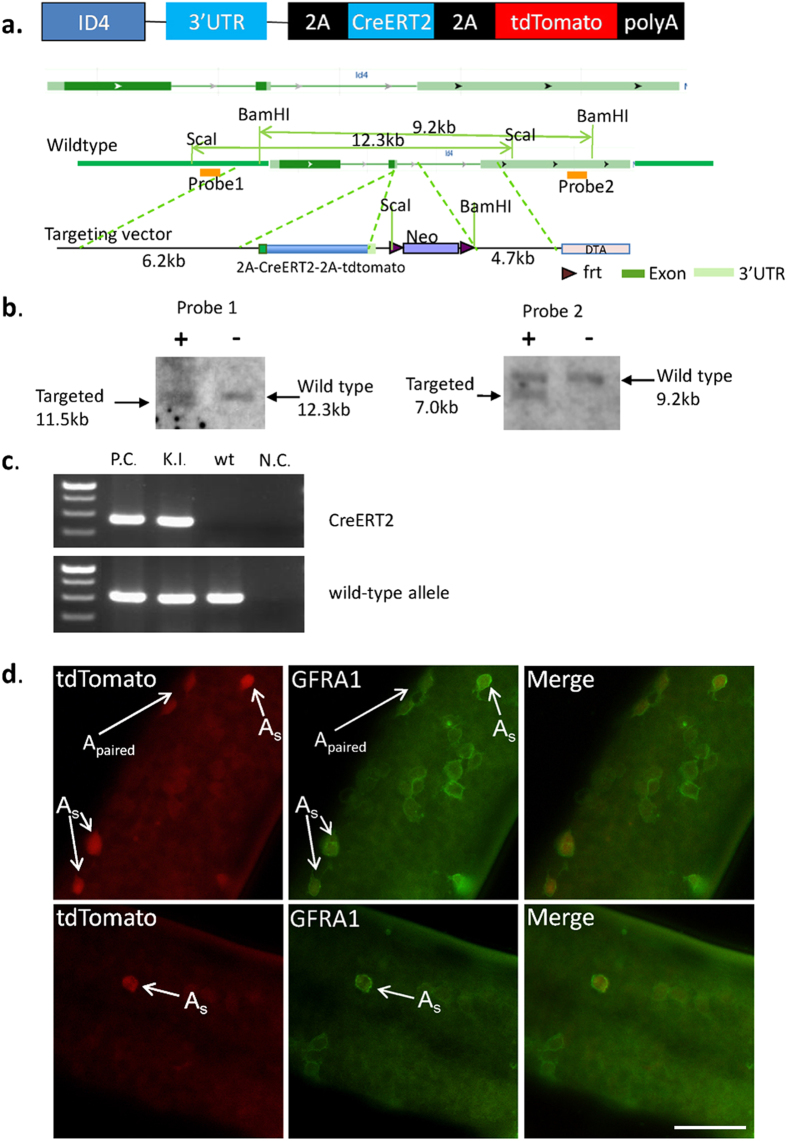
Generation of Id4-2A- CreERT2-2A-tdTomato knock-in mice. (**a**) Targeting strategy to generate Id4-2A-CreERT2-2A-tdTomato knock-in mice. Restriction sites, Southern blot analysis probes, and expected restriction fragment lengths are indicated. UTR, untranslated regions; 2A, 2A peptide (identified among picornaviruses and results in the cotranslational “cleavage” of proteins); CreERT2, a tamoxifen inducible Cre-estrogen receptor (ER) fusion protein. (**b**) Southern blot analysis to confirm correct integration of Id4-CreERT2-2A-tdTomato allele in ESCs. Southern blot analysis of ESC clone demonstrates the successful generation of the targeted Id4-tdTomato-2A-CreERT2 allele. (**c**) Genotyping of wild-type and heterozygous mice by PCR using primer pairs specific to the Id4-CreERT2-tdTomato allele (CreERT2) and wild-type allele. P.C., positive control; K.I., Id4-CreERT2-tdTomato knock-in mice; wt, wild-type mice; N.C., negative control. (**d**) Fluorescence image of whole mount staining of testes from Id4 -creERT2-2A-tdToamto mice. Immunofluorescence staining for GFRA1 shows the tdTomato is expressed in A_single_ and A_paired_ GFRA1^+^ undifferentiated spermatogonia in testes of Id4 knock-in mice. Scale bar, 50 μm.

Homologous recombination was used to generate embryonic stem-cell (ESC) clones in which a 2A-CreERT2-2A-tdTomato cassette was inserted into the 3′ UTR of the Id4 allele (Fig. 1a). This 2A-CreERT2-2A-tdTomato expression cassette ultimately generates CreERT2 and tdTomato proteins in Id4^+^ cells without disrupting Id4 expression. Positive ESC clones were isolated after selection with G418 and confirmed by Southern blot analysis (Fig. 1b). A PCR method for genotyping was used to verify the structure of the targeted allele and to identify Id4-CreERT2-tdTomato mice (Fig. 1c).

**Figure 2:**
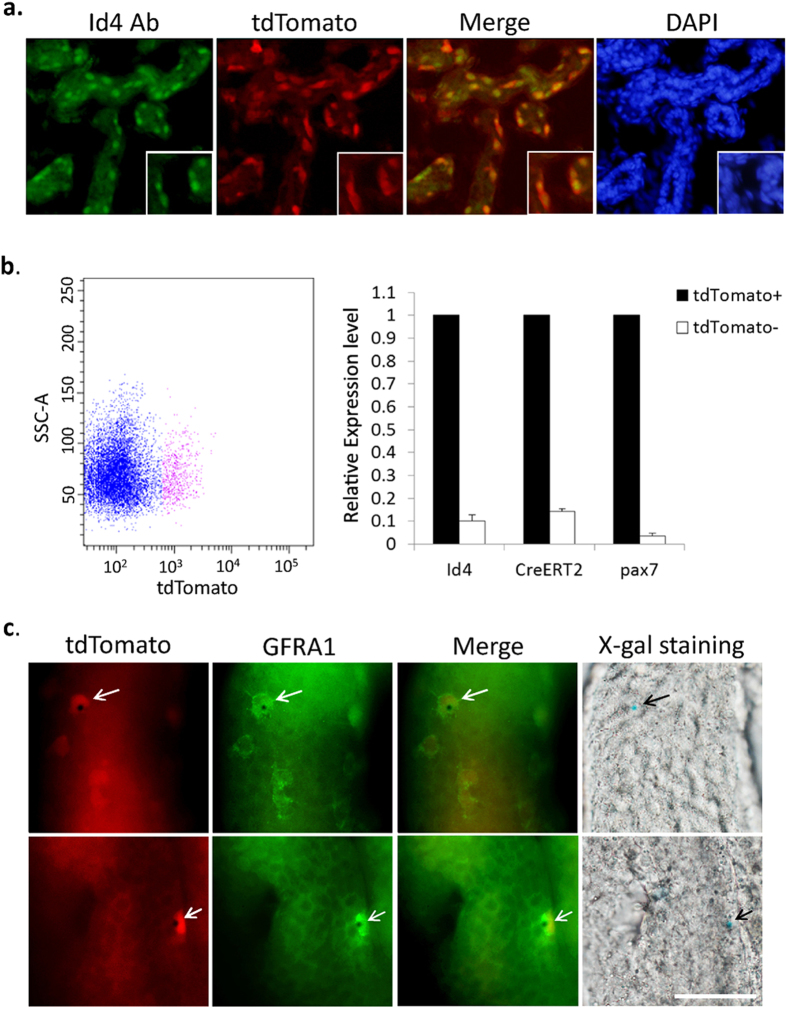
Identification of Id4-2A-CreERT2-2A-tdTomato knock-in mice. (**a**) Fluorescence image of frozen sections of Id4-creERT2-tdTomato mouse testes. Immunofluorescence staining shows the Id4 protein was coexpressed with tdTomato in the testes of Id4 knock-in mice. (**b**) Left panel, Representative scatter plots of tdTomato^+^ and tdTomato^−^ populations isolated from the testes of 8-day-old Id4 knock-in mice(n = 5), determined using fluorescence-activated cell sorting (FACS). Right panel, The levels of Id4, Cre and Pax7 mRNAs were determined by real-time PCR in the tdTomato^+^ population compared with tdTomato^−^ population. The value from the tdTomato^+^ sample was set as 1. GADPH served as an internal control. (**c**) Expression of lacZ is detected only in Id4^+^ cells of Id4-CreERT2-tdTomato; Rosa26- flox-stop-LacZ mice using X-gal staining, 2 days after TM induction. Whole mount staining for GFRA1 after X-gal staining showed that these lacZ^+^ cells are also GFRA1^+^ undifferentiated spermatogonia. Scale bar, 50 μm.

To validate the transgene expression patterns of the Id4-CreERT2-tdTomato knock-in alleles, we examined tdTomato expression in the mouse testis. Consistent with a previous study, tdTomato expression was observed in A_single_ cells, and A_paired_ cells (cysts of interconnected cell pairs; 24 tdTomato^+^ A_paired_ cells were observed out of 153 tdTomato^+^ cells) (Fig. 1d). Results of coimmunofluorescence staining with GFRA1 revealed that the Id4-tdTomato^+^ undifferentiated spermatogonial population represents a subset of the GFRA1^+^ undifferentiated spermatogonial population (Fig. 1d, Supplemental Fig. 1a). Immunofluorescence staining showed the Id4 protein was co-expressed with tdTomato in the undifferentiated spermatogonial population in the testis of Id4 knock-in mice (Fig. 2a, Supplemental Fig. 1b.). Next, fluorescence-activated cell sorting (FACS) was performed on the basis of tdTomato to isolate tdTomato^+^ and tdTomato^–^ cells from the testes of 8-day-old Id4 knock-in mice. We found that Id4, CreERT2, and the SSC marker pax7 are highly expressed in tdTomato^+^ cells, compared with tdTomato^−^ cells (Fig. 2b). These observations confirmed that tdTomato and CreERT2 are expressed in Id4^+^ cells. We further examine CreERT2 expression by using Id4-creERT2-tdTomato; ROSA26-lacZ mice. Two days after tamoxifen (TM) injection, expression of lacZ was detected in only the Id4^+^ cells of Id4-CreERT2-tdTomato;ROSA26-flox-stop- LacZ mice using X-gal staining 2 days after TM administration (Fig. 2c). Whole mount staining for GFRA1 after X-gal staining showed that these lacZ-expressing (lacZ^+^) cells are also GFRA1^+^ undifferentiated spermatogonia (Fig. 2c). Activation of Cre and tdTomato do not influence normal spermatogenesis. Thus, the Id4-CreERT2-tdTomato knock-in alleles faithfully document endogenous Id4 expression, at least in the undifferentiated spermatogonial population.

### Id4^+^ Cells Contribute to the Differentiation and Self-Renewal in Mouse Spermatogenesis

**Figure 3:**
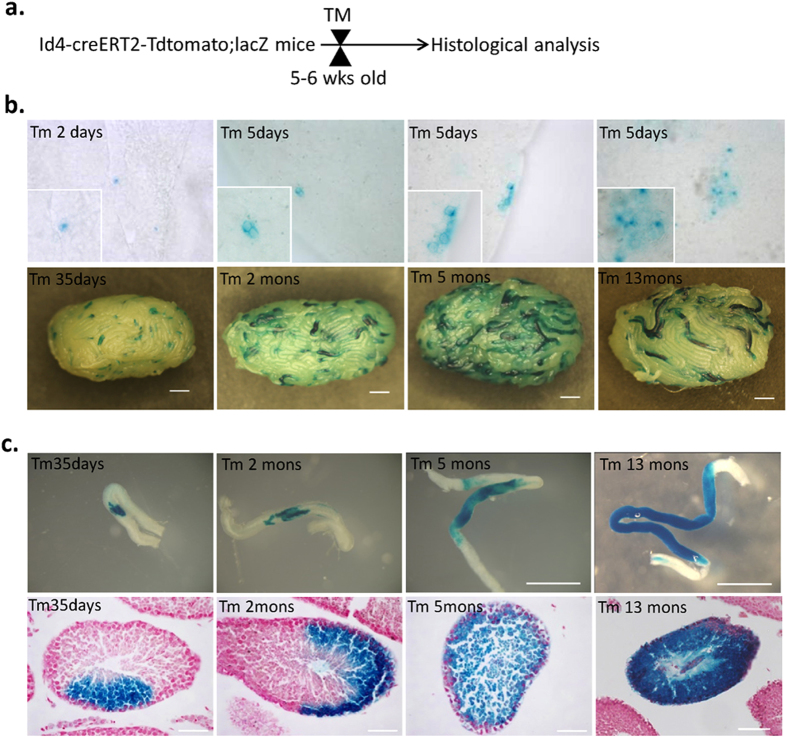
Id4^+^ Cells Contribute to Differentiation and Self-Renewal in mouse spermatogenesis. (**a**) Experimental strategy for lineage tracing. (**b**) Whole-mount seminiferous tubule X-gal staining of Id4-CreERT2-tdTomato; ROSA26-flox-stop lacZ adult mice at various time points after a single TM injection. Scale Bar, 1 mm. (**c**) Top panel, Representative image of clones of LacZ^+^ cells. Scale bar, 1 mm. Bottom panel, X-gal-stained (blue) sections counterstained with neutral red (pink). Scale bar, 50 μm.

In order to permanently label Id4^+^ cells and their progeny *in vivo*, we initially injected TM intraperitoneally into 5- to 6-week-old Id4-CreERT2-tdTomato; ROSA26-flox-stop-lacZ mice. LacZ expression in the testis was examined at various time points after TM injection (Fig. 3a). No marking was noted in wild-type mice that were treated with TM (Supplemental Fig. 2a) or in Id4-CreERT2-tdTomato; ROSA26-flox-stop-lacZ double transgenic mice that were treated with vehicle, suggesting that the CreERT2 constructs are not “leaky” without tamoxifen treatment (Supplemental Fig. 2b). Two days after TM treatment, most lacZ^+^ clones were A_single_ cells or A_paired_ cells in rare incidences (n = 5 mice). Five days after tamoxifen treatment, we could find lacZ^+^ A_paired_, and A_aligned_ (interconnected cells in syncytial cysts of 4, 8, 16, and occasionally 32 cells) cells (n = 5 mice). The lacZ^+^ clones were persisted and larger at 35 days (n = 6 mice), 2 months (n = 5 mice), 5 months (n = 5mice), and 13 months (n = 9 mice) after TM injection (Fig. 3b). The tubule section was sometimes entirely filled with LacZ^+^ cells, demonstrating production of all stages of male germ cells by Id4^+^ SSCs. This result indicates that each clone was derived from a labeled Id4^+^ cell that had continuously produced differentiating cells while self-renewing for at least one complete round of spermatogenesis (Fig. 3c). Taken together, our observations suggest that Id4^+^ cells are capable of self-renewal and have differentiation potential.

### Id4 Marks Spermatogonial Stem Cells

**Figure 4:**
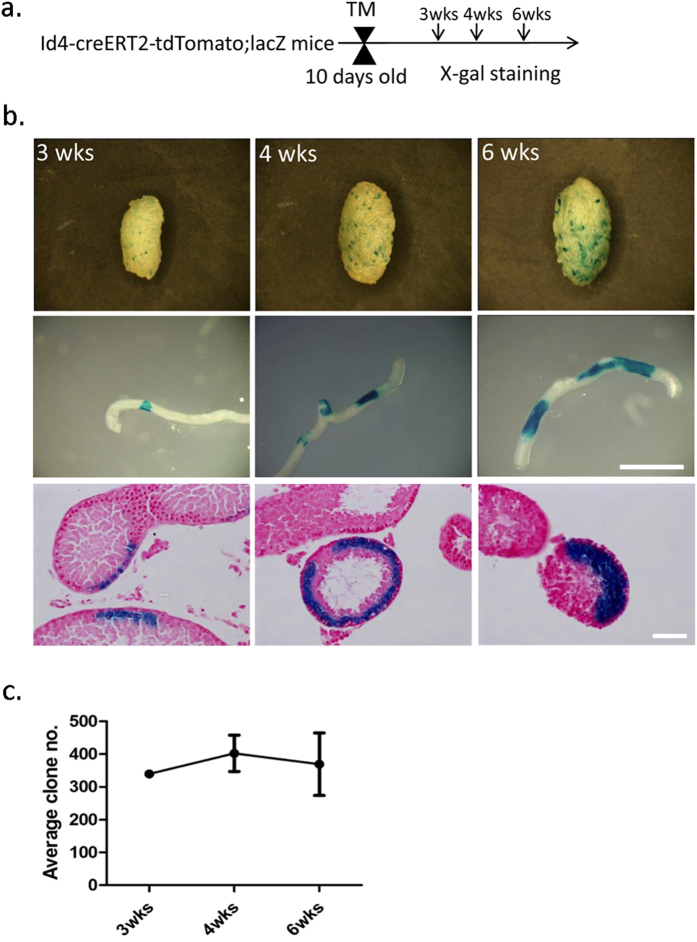
Id4 Marks SSCs. (**a**) The experimental schedule. Id4-creERT2-tdTomato;ROSA26-flox-stop-lacZ mice were treated with TM once at 10 days of age. X-gal staining was performed at 3 weeks, 4 weeks, and 6 weeks after TM induction. (**b**) Upper row, Whole-mount seminiferous tubule X-gal staining of Id4-CreERT2-tdTomato; Rosa-lacZ mice. Middle row, Clones of LacZ-positive cells at 3 weeks, 4 weeks, and 6 weeks after TM induction. Scale bar, 1 mm. Lower row, Cross-sections of the clones at 3 weeks, 4 weeks, and 6 weeks after TM induction. X-gal-stained (blue) sections counterstained with neutral red (pink). Scale bar, 50 μm. (**c**) Average clone numbers at 3 weeks,4 weeks, and 6 weeks after TM induction in testes (n = 4). The average clone number remained constant over time.

To examine whether Id4 marks SSCs, we labeled Id4^+^ cells and their progeny by intraperitoneal injection of TM into a cohort of 10-day-old Id4-CreERT2-tdTomato; ROSA26-flox-stop-lacZ mice. The testes were isolated and stained with X-gal 3 weeks, 4 weeks or 6 weeks after the TM treatment (Fig. 4a). The number of lacZ^+^ clone was determined. We found that the average clone size increased over time. However, the average clone number remained stable (Fig. 4b,c).This suggests that Id4^+^ spermatogonia are spermatogonial stem cells. Because 6 weeks is longer than the total duration of mouse spermatogenesis, if the labeled Id4^+^ spermatogonia are not SSCs, labeled cells wound differentiate into sperm and eventually disappear; thus, the clone number would decrease.

### Depletion Experiments Demonstrate that Id4^+^ Cells Are Necessary for Spermatogenesis

**Figure 5:**
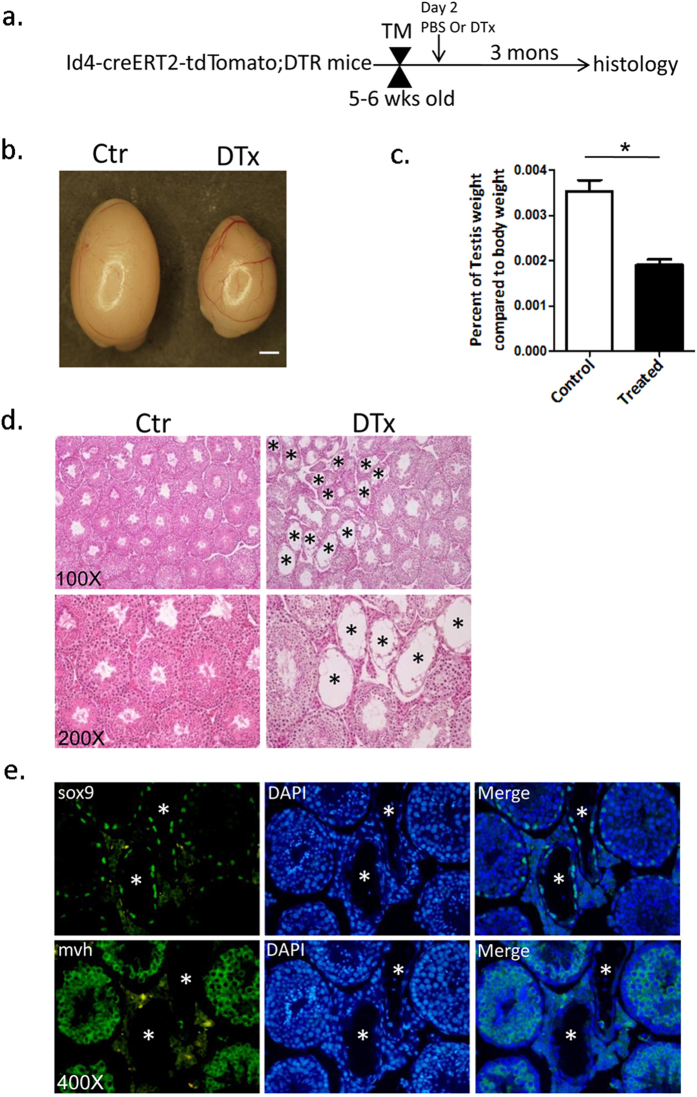
Ablation of Id4^+^ cells in mice results in a disruption of spermatogenesis. (**a**) Experimental strategy for DTx treatment. (**b**) Intact testes from PBS-treated (Ctr) and DTx-treated mice (DTx). Id4-CreERT2-tdTomato;ROSA26-flox-stop-DTR mice were treated with PBS or DTx after TM induction. The testes of Id4-CreERT2-tdTomato;ROSA26-DTR mice appeared smaller in size after 3 months of DTx treatment. Scale bar, 1 mm. (**c**) Comparison of testes weights between control mice (Ctr) and DTx-treated Id4-CreERT2-tdTomato;ROSA26-DTR mice (DTx) (n = 7). *Denotes significant difference at P = 1.01133E-05. Statistical significance was determined using a two-tailed Student’s t-test. (**d**) H&E staining of paraffin-embedded sections from the testis of Id4-CreERT2-tdTomato;ROSA26-flox-stop-DTR mice that received a single tamoxifen with PBS treatment(Ctr, left column) or with single DTx treatment(DTx,right column). There are more degenerated tubules (*) in DTx-treated mice (right column) than in control mice (left column). (**e**) Imunofluorescence staining for Sox9 and Mvh confirmed the almost complete absence of germ cells in some tubules of DTx-treated Id4-CreERT2-tdTomato;ROSA26-DTR mice with very few germ cells remaining. The Mvh-stained cells are germ cells. The cells stained with Sox9 are Sertoli cells. Original magnifications are as indicated. *degenerated tubules.

To determine the physiological requirement for Id4^+^ cells during spermatogenesis, we performed targeted ablation of these cells in the mouse testis. We generated Id4-CreER-tdTomato;ROSA26-flox-stop-DTR mice to conditionally express diphtheria toxin receptor(DTR) in Id4^+^ cells. The Id4^+^ cells were depleted following administration of diphtheria toxin (DTx) after TM induction (Fig. 5a). Three months after DTx treatment, the testes of Id4-CreERT2-tdTomato;ROSA26-DTR mice appeared smaller in size and lighter in weight than those of PBS-treated control mice (Fig. 5b,c). Consistent with this, histological analysis showed an increased number of degenerated tubules in the testes of DTx-treated mice (Fig. 5d). Imunofluorescence staining for Sox9 and Mvh confirmed that only sertoli cells left in some tubules of DTx-treated Id4-CreERT2-tdTomato;ROSA26-DTR mice with very few germ cells remaining (Fig. 5e). As an additional control, we found that spermatogenesis was not affected in wild-type mice treated with TM and DTx (Supplemental Fig. 3). Together, these results suggest that Id4^+^ germ cells are essential for mouse spermatogenesis and depletion of Id4^+^ cells results in impaired spermatogenesis. This is also consistent with our previous result that Id4 marks SSCs in mouse testes.

### Contribution of the Pulse-Labeled Id4^+^ SSCs to Regeneration

**Figure 6:**
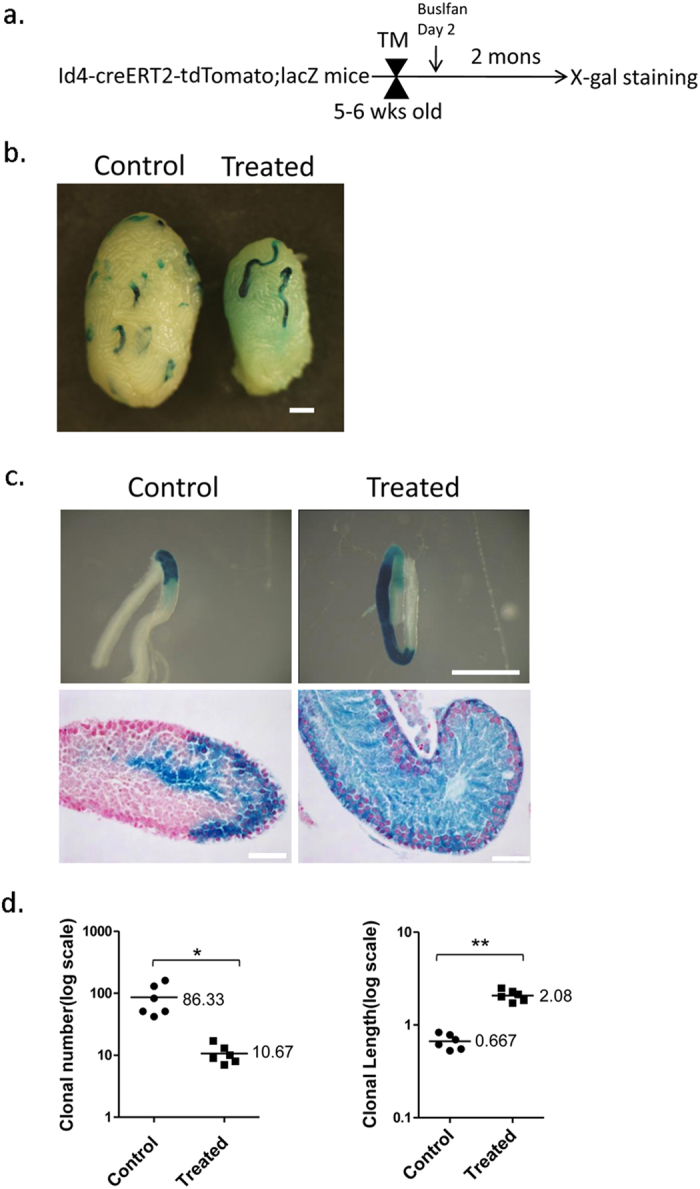
Contribution of the Pulse-Labeled Id4^+^ SSCs to Regeneration. (**a**) The experimental schedule. Id4-CreERT2-tdTomato;ROSA26-flox-stop-lacZ double transgenic mice were TM pulsed at 5-6 weeks of age. Busulfan (10 mg/kg) was injected to induce regeneration after 2 days. Two months later, the contribution of the pulse-labeled Id4^+^ spermatogonia to the regenerating spermatogenesis was evaluated after X-gal staining, and then compared with controls that were treated in the same manner, but without busulfan injection. (**b**). Whole-mount seminiferous tubule X-gal staining of busulfan-treated (Treated) and untreated Id4-CreERT2-tdTomato;ROSA26-lacZ mice (Control). Scale bar, 1 mm. (**c**) Top panel, Clones of LacZ-positive cells with (Treated, right panel) or without (Control, left panel) busulfan treatment. Scale bar, 1 mm. Bottom panel, Cross-sections of the clones with (Treated, right panel) or without (Control, left panel) busulfan treatment. X-gal-stained (blue) sections counterstained with neutral red (pink). Scale bar, 50 μm. (**d**) Left panel, Number of LacZ^+^, labeled cell-derived clones with (Treated, n = 6 mice) or without (Control, n = 6 mice) busulfan treatment. The clone number is decreased in testes treated with busulfan. Right panel, Length of LacZ^+^, labeled cell-derived clones with (Treated, n = 6) or without (Control, n = 6) busulfan treatment. The clone length is increased in testes treated with busulfan treatment compared with untreated mice. Average numbers and length of clones per testis are shown. *P = 0.003605204, **P = 5.63421E-07 (two-tailed Student’s t-test).

Next, the contribution of Id4^+^ spermatogonia to regeneration was investigated. As shown in Figure 6a, 5–6-week-old Id4-creERT2-tdTomato;ROSA26- flox-stop-lacZ mice were pulse-labeled with TM, and treated with 10 mg/kg busulfan to induce regeneration. Two months later, the testes were isolated and stained with X-gal. As expected, the testes treated with busulfan underwent massive germ cell death and appeared smaller in size (Fig. 6b). The mean number of Plzf^+^ undifferentiated spermatogonia per tubule cross-section was significantly decreased 8 days after busulfan treatment, compared with that of controls (Supplemental Fig. 4).

The contribution of the pulse-labeled Id4^+^ spermatogonia to regenerating spermatogenesis was evaluated after X-gal staining. The clone number was decreased in testes of mice treated with busulfan, compared with those of the controls, which were treated in the same manner without the busulfan injection; however, the length of the clones in treated mice was greater than in untreated controls (Fig. 6c,d). A total of 86.33 ± 19.96 (n = 6) lacZ^+^ clones were detected in untreated mice after 2 month, whereas 10.67 ± 1.52 (n = 6) lacZ^+^ clones were detected in treated mice. Although the clone number is fewer in treated testes than in untreated testes, cross-sections of lacZ^+^ clones in treated mice show that the labeled cells contain all stages of male germ cells. These results suggest that Id4^+^ SSCs contribute to full-lineage maturation after busulfan treatment, as in normal spermatogenesis, in untreated mice (Fig. 6d). The length of clones in untreated mice was 0.667 ± 0.05 mm, whereas the clone length in treated mice was 2.08 ± 0.116 mm. Examination of the clone length provides an assessment of the capacity for proliferative expansion^6^,^12^,^13^. The clone length was increased in the treated mice, which means that Id4^+^ SSCs expand during regeneration upon injury. Thus, Id4^+^ SSC expansion accounts for the increased clone length. This also demonstrates that Id4^+^ SSCs play an important role during spermatogenic regeneration. Taken together, these data demonstrate that Id4^+^ spermatogonia contribute to the recovery of spermatogenesis after busulfan insult to the germ cells.

### Id4^+^ SSCs Originate from Neonate Id4^+^ Gonocytes

**Figure 7:**
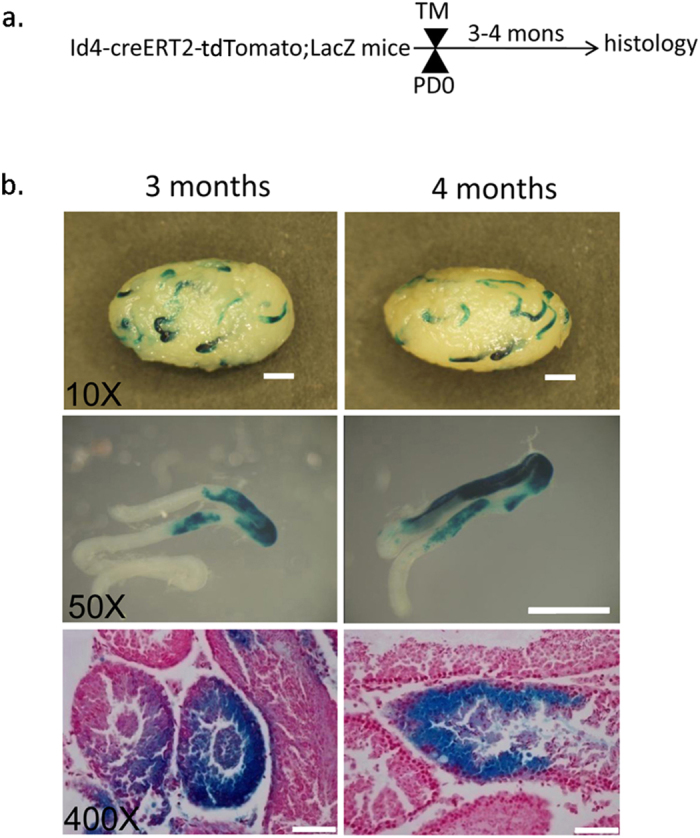
Id4^+^ SSCs originate from neonate Id4^+^ gonocytes. (**a**) Experimental design. (**b**) Upper row, Whole-mount LacZ staining. Time course of cell lineage mapping experiments for postnatal stage. Scale bar, 1 mm. Middle row, Clones of LacZ^+^ cells at 3 months (n = 5) and 4 months (n = 5) after TM induction. Scale bar, 1 mm. Lower row, Cross-sections of the clones at 3 months and 4 months after TM induction. X-gal-stained (blue) sections counterstained with neutral red (pink). Scale bar, 50 μm.

Given that Id4 is already expressed in most gonocytes at Postnatal Day (PD) 0^31^, we wondered whether Id4^+^ gonocytes are the precursors of Id4^+^ SSCs. To determine this, we injected Id4-CreERT2-tdTomato; ROSA26-flox-stop-lacZ mice with TM at PD0 and analyzed their testes as adults (Fig. 7a). Similar to results obtained with adult labeling, lacZ^+^ clones were detected in the testes at 3 months and 4 months after TM induction (Fig. 7b). Labeled clones contained immature spermatogonia and mature sperm (Fig. 7b). Developmental fate mapping revealed that Id4^+^ SSCs originate from neonate Id4^+^ gonocytes.

## Discussion

Our lineage-tracing experiments using Id4-CreERT2-tdTomato;ROSA26- flox-stop-lacZ mice showed that labeled Id4^+^ spermatogonia persist in the testis and continue to produce differentiating progeny for 13 months. These results indicate that Id4 marks SSCs in mouse testis. Previous studies have shown that expression of Id4 is selective for A_single_ cells within the spermatogonial population of mouse testes, and that Id4^+^ cells have regenerative capacity in transplantation experiments. These studies, together with our results, suggest that Id4 is a marker of a subset of A_single_ SSCs. Recently, Pax7 has been identified and characterized by Aloisio *et al.*, as a new marker for a subset of A_single_ SSCs in the mouse testis^9^. Our results showed that Pax7 is more highly expressed in tdTomato^+^ cells than in tdTomato^–^ cells, which suggests that Pax7 and Id4 are expressed in overlapping subsets of A_single_ spermatogonia. These findings indicate that the A_single_ spermatogonial population in the mouse testes is heterogeneous^31^,^32^,^33^,^34^.

In the scheme of Huckins^35^ and Oakberg^36^, the A_single_ cells among the undifferentiated A spermatogonia are the SSCs, implying that the formation of a pair constitutes the first step of differentiation. In theory, however, not all A_single_ cells can be stem cells, as some A_single_ cells that form A_paired_ cells eventually become spermatozoa^37^. Our lineage-tracing experiment showed that the labeled clone number is constant when Id4^+^ cells are labeled at PD10, which suggests that most Id4^+^ spermatogonia, if not all, are SSCs. During the last decade, the A_s_ model has been challenged. It has been suggested that not all A_single_ spermatogonia are SSCs, and that pairs and chains can split up during steady-state spermatogenesis, thus providing new single SSCs, pairs, and shorter chains in an alternative way^4^,^5^,^38^,^39^,^40^. A recent study showed that the entire GFRA-1^+^ population comprises a single stem-cell pool, in which cells continually interconvert between A_s_ and syncytial states during steady-state spermatogenesis and post-insult regeneration^8^. These observations suggest that the stem-cell pool might be not restricted to A_single,_ and might be expanded to include A_paired_ and A_aligned_. Future investigations are needed to elucidate the cellular hierarchies underlying stem-cell maintenance and differentiation in the testis.

Here, we observed tdTomato expression in A_single_ spermatogonia, A_paired_ spermatogonia, and some spermatocytes at around PD20.Similarly, Oatley’s team observed that GFP expression in Id4-GFP transgenic mice was detected in both a subset of type A spermatogonia and some pachytene spermatocytes at PD20 and PD35 but not in other spermatogonial subtypes, other spermatocytes, or spermatids^31^. Furthermore, a previous study showed that immunostaining for the expression of endogenous Id4 did not reveal the presence of Id4 in pachytene spermatocytes^30^. We also examined the expression of endogenous Id4 by using immunofluorescence analysis. Staining for Id4 expression was observed in type A spermatogonia, but we did not observe obvious staining in pachytene spermatocytes. It is still possible that Id4 expression is also present in spermatocytes. However, the level of Id4 expression may be too low to be detected in our immunofluorescence analysis. Regardless, our study demonstrated that expression of the Id4-creERT2-tdTomato transgene is specifically restricted to A_single_ spermatogonia and some A_paired_ spermatogonia within the undifferentiated spermatogonial population of mouse testes. More importantly, the Id4^+^ spermatogonia persisted in the testis and continued to give rise to differentiating progeny for 13 months, indicating that the population has long-term stem-cell potential *in vivo*.

In previous reports, many more labeled clones served by a labeled SSC were found in Ngn3-creER;Rosa-lacZ mice that received busulfan than were found in mice that did not receive busulfan^5^. Ngn3-creER labeled potential SSCs. The killing of some SSCs triggered a number of “potential” SSCs to become “actual” SSCs that subsequently self-renewed and formed clones. So the clone number increases when mice are treated with busulfan. In our study, fewer clones were detected in busulfan-treated mice, because Id4 marks actual stem cells, and no potential stem cells become actual stem cells. However the clone length is increased in busulfan-treated mice, which suggests that the SSCs expand during regeneration. Our results are consistent with those of a recent study^9^. In that study, PAX7^+^ spermatogonia expansion contributed to the recovery of spermatogenesis in mice subjected to chemotherapy and radiotherapy. SSC expansion might be one of the mechanisms by which SSCs respond to injury.

Oatley’s study demonstrated that Id4-Gfp expression marks spermatogonia that possess the core ability to function as a SSC in SSC transplantation experiments^31^. However, it is unknown whether the actual stemness could have been observed if the cells had been studied during steady-state spermatogenesis. Our lineage-tracing experiments revealed that Id4^+^ cells continuously generate all stages of germ cells in the undisturbed testis and during regenerating spermatogenesis, documenting their self-renewal ability and differentiation potential. Our study provides information complementary to the previous reports from Oatley’s group. Their studies, together with our results, suggest that Id4 marks spermatogonia stem cells in the mouse testis.

In summary, Id4 marks a rare but functionally important SSC population during steady-state conditions, and also play an important role in spermatogenic regeneration following injury to germ cells.

## Materials And Methods

### Mice

All mice were maintained under a 12-hour light/dark cycle. Id4-2A-CreERT2 -2A-tdTomato knock-in mice were generated by Beijing Biocytogen Co., Ltd. Briefly, C57BL/6 ESCs were targeted with knock-in constructs containing 2A-CreERT2-2A-tdTomato under the control of endogenous Id4 regulatory elements using standard protocols. Correct insertion of constructs was verified by Southern blotting, and correctly targeted clones were injected into blastocysts and transferred into pseudopregnant females. The resulting chimeric mice were bred with C57/BL6 mice and their germline offspring were bred to establish stable lines. Experimental procedures were approved by the Animal Care and Use Committee of Shanghai Institute of Biochemistry and Cell Biology, Chinese Academy of Sciences, and all methods were carried out in accordance with the guidelines approved by the Animal Care and Use Committee of Shanghai Institute of Biochemistry and Cell Biology, Chinese Academy of Sciences.

Id4 knock-in mice were maintained as homozygotes. ROSA26-flox-stop-LacZ mice (Gt(ROSA)26Sortm1Sho/J)^41^, in which LacZ is a reporter for Cre recombination, were also maintained as homozygotes. ROSA26-flox-stop-DTR mice, in which GFP and diphtheria toxin receptor (DTR) are expressed when Cre is activated, were kindly provided by Beijing Biocytogen Co., Ltd. Id4-CreERT2-tdTomato mice were crossed with the ROSA26-flox-stop-LacZ (or DTR) reporter mice to generate the Id4-CreERT2-tdTomato/+;ROSA26-flox-stop- LacZ (or DTR)/+mice used in this study. In these animals, TM administration is expected to transiently activate Cre in a subset of Id4^+^ cells, resulting in the permanent expression of LacZ (or DTR) in those cells and their descendants. For genotyping, genomic DNA was extracted from tail tips and assayed using polymerase chain reaction (PCR) primer sets for the Rosa26R allele (F: AATCCATCTTGTTCAATGGCCGATC, R: CCGGATTGATG

GTAGTGGTC), for the Id4-CreER allele (F: TGATATGCGCACTCTAACCGT, R:CGA

TCCCTGAACATGTCCATCAG), and for the DTR allele (F: GCATCGATACCGTCGACCTC, R: GCTGTCCATCTGCACGAGAC).

### Tamoxifen Injection

Id4 knock-in males were crossed with ROSA26-flox-stop-LacZ (or DTR) females. TM was dissolved in corn oil (10 mg/ml). Mice were treated with intraperitoneal injections of TM at approximately 5–6 weeks of age.

### X-gal Staining

Whole-mount X-gal staining was performed by manually dissociating tubules, fixing them in 4% paraformaldehyde for 2 hours at 4 °C, and staining in the dark in standard β-galactosidase substrate (5 mM potassium ferricyanide, 5 mM potassium ferrocyanide, 1 mg/ml X-gal, 2 mM MgCl_2_, 0.01% sodium deoxycholate, 0.02% NP-40 in PBS) overnight at room temperature, as previously described^42^. Photographic images were then obtained by using an Olympus SZ16 light microscope.

### Histology and Immunohistochemistry

Testes were fixed in 4% paraformaldehyde or Bouin’s solution. Tissue sections were stained with hematoxylin and eosin for H&E visualization. For immunofluorescence staining, sections were permeabilized with 0.5% Triton X-100 for 10 minutes and blocked in 0.1% Triton-PBS, 0.25% goat normal serum for 1 hour at room temperature. Primary antibodies were incubated overnight at 4 °C and washed with PBS. Secondary antibodies were incubated for 1 hour at room temperature. Antibodies used were as follows: Id4 (CalBioreagents), Mvh (Abcam), and Sox9 (EMD Millipore).

### RNA isolation and Quantitative Real-Time PCR

Total RNA was isolated from the cells by using Trizol, followed by reverse transcription. Quantitative real-time PCR was performed using the SYBR Green Master Mix (Toyobo) on a Real-Time PCR Detection System (Eppendorf). For the reverse transcription reaction 1 μg of total RNA was used. Expression levels were calculated by using the comparative CT method with GAPDH as an endogenous reference gene.

### FACS Analyses

Dissociated cells from Id4 knock-in mice were suspended in DMEM/10% FBS. FACS analyses were performed by using an Aria II cell sorter (BD Biosciences, San Jose, CA).

### Statistics

Statistics were calculated using GraphPad software. Error bars in all figures indicate SEM for at least 3 animals. Differences resulting in P value < 0.05 were considered significant.

## Additional Information

**How to cite this article**: Sun, F. *et al.* Id4 Marks Spermatogonial Stem Cells in the Mouse Testis. *Sci. Rep.* **5**, 17594; doi: 10.1038/srep17594 (2015).

## Supplementary Material

Supplementary Information

## References

[b1] Weissman I. L. Translating stem and progenitor cell biology to the clinic: barriers and opportunities. Science 287, 1442–1446 (2000). 10688785 10.1126/science.287.5457.1442

[b2] Yoshida S. Elucidating the identity and behavior of spermatogenic stem cells in the mouse testis. Reproduction 144, 293–302, 10.1530/REP-11-0320 (2012). 22733803

[b3] Nagano M. C. & Yeh J. R. The identity and fate decision control of spermatogonial stem cells: where is the point of no return? Current topics in developmental biology 102, 61–95, 10.1016/B978-0-12-416024-8.00003-9 (2013). 23287030

[b4] Nakagawa T., Sharma M., Nabeshima Y., Braun R. E. & Yoshida S. Functional hierarchy and reversibility within the murine spermatogenic stem cell compartment. Science 328, 62–67, 10.1126/science.1182868 (2010). 20299552 PMC2981100

[b5] Nakagawa T., Nabeshima Y. & Yoshida S. Functional identification of the actual and potential stem cell compartments in mouse spermatogenesis. Developmental cell 12, 195–206, 10.1016/j.devcel.2007.01.002 (2007). 17276338

[b6] Klein A. M., Nakagawa T., Ichikawa R., Yoshida S. & Simons B. D. Mouse germ line stem cells undergo rapid and stochastic turnover. Cell stem cell 7, 214–224, 10.1016/j.stem.2010.05.017 (2010). 20682447

[b7] Sada A., Suzuki A., Suzuki H. & Saga Y. The RNA-binding protein NANOS2 is required to maintain murine spermatogonial stem cells. Science 325, 1394–1398, 10.1126/science.1172645 (2009). 19745153

[b8] Hara K. *et al.* Mouse spermatogenic stem cells continually interconvert between equipotent singly isolated and syncytial states. Cell stem cell 14, 658–672, 10.1016/j.stem.2014.01.019 (2014). 24792118 PMC4010676

[b9] Aloisio G. M. *et al.* PAX7 expression defines germline stem cells in the adult testis. The Journal of clinical investigation 124, 3929–3944, 10.1172/JCI75943 (2014). 25133429 PMC4153705

[b10] Brinster R. L. & Avarbock M. R. Germline transmission of donor haplotype following spermatogonial transplantation. Proceedings of the National Academy of Sciences of the United States of America 91, 11303–11307 (1994). 7972054 10.1073/pnas.91.24.11303 PMC45219

[b11] Brinster R. L. & Zimmermann J. W. Spermatogenesis following male germ-cell transplantation. Proceedings of the National Academy of Sciences of the United States of America 91, 11298–11302 (1994). 7972053 10.1073/pnas.91.24.11298 PMC45218

[b12] Nagano M., Avarbock M. R. & Brinster R. L. Pattern and kinetics of mouse donor spermatogonial stem cell colonization in recipient testes. Biology of reproduction 60, 1429–1436 (1999). 10330102 10.1095/biolreprod60.6.1429 PMC5511737

[b13] Dobrinski I., Ogawa T., Avarbock M. R. & Brinster R. L. Computer assisted image analysis to assess colonization of recipient seminiferous tubules by spermatogonial stem cells from transgenic donor mice. Molecular reproduction and development 53, 142–148, 10.1002/(SICI)1098-2795(199906)53:2<142::AID-MRD3>3.0.CO;2-O(1999 ). 10331452

[b14] Kanatsu-Shinohara M. *et al.* Clonal origin of germ cell colonies after spermatogonial transplantation in mice. Biology of reproduction 75, 68–74, 10.1095/biolreprod.106.051193 (2006). 16598026

[b15] Zhang X., Ebata K. T. & Nagano M. C. Genetic analysis of the clonal origin of regenerating mouse spermatogenesis following transplantation. Biology of reproduction 69, 1872–1878, 10.1095/biolreprod.103.019273 (2003). 12904317

[b16] Barker N. *et al.* Identification of stem cells in small intestine and colon by marker gene Lgr5. Nature 449, 1003–1007, 10.1038/nature06196 (2007). 17934449

[b17] Barker N., Bartfeld S. & Clevers H. Tissue-resident adult stem cell populations of rapidly self-renewing organs. Cell stem cell 7, 656–670, 10.1016/j.stem.2010.11.016 (2010). 21112561

[b18] Snippert H. J. & Clevers H. Tracking adult stem cells. EMBO reports 12, 113–122, 10.1038/embor.2010.216 (2011). 21252944 PMC3049439

[b19] Barker N. *et al.* Lgr5(+ve) stem cells drive self-renewal in the stomach and build long-lived gastric units *in vitro*. Cell stem cell 6, 25–36, 10.1016/j.stem.2009.11.013 (2010). 20085740

[b20] Ling F., Kang B. & Sun X. H. Id proteins: small molecules, mighty regulators. Current topics in developmental biology 110, 189–216, 10.1016/B978-0-12-405943-6.00005-1 (2014). 25248477

[b21] Nam H. S. & Benezra R. High levels of Id1 expression define B1 type adult neural stem cells. Cell stem cell 5, 515–526, 10.1016/j.stem.2009.08.017 (2009). 19896442 PMC2775820

[b22] Yun K., Mantani A., Garel S., Rubenstein J. & Israel M. A. Id4 regulates neural progenitor proliferation and differentiation *in vivo*. Development 131, 5441–5448, 10.1242/dev.01430 (2004). 15469968

[b23] Samanta J. & Kessler J. A. Interactions between ID and OLIG proteins mediate the inhibitory effects of BMP4 on oligodendroglial differentiation. Development 131, 4131–4142, 10.1242/dev.01273 (2004). 15280210

[b24] Park H. J. *et al.* Elevated Id2 expression results in precocious neural stem cell depletion and abnormal brain development. Stem cells 31, 1010–1021, 10.1002/stem.1351 (2013). 23390122 PMC3637429

[b25] Jankovic V. *et al.* Id1 restrains myeloid commitment, maintaining the self-renewal capacity of hematopoietic stem cells. Proceedings of the National Academy of Sciences of the United States of America 104, 1260–1265, 10.1073/pnas.0607894104 (2007). 17227850 PMC1783103

[b26] Perry S. S. *et al.* Id1, but not Id3, directs long-term repopulating hematopoietic stem-cell maintenance. Blood 110, 2351–2360, 10.1182/blood-2007-01-069914 (2007). 17622570 PMC1988946

[b27] Junankar S. *et al.* ID4 controls mammary stem cells and marks breast cancers with a stem cell-like phenotype. Nature communications 6, 6548, 10.1038/ncomms7548 (2015). 25813983

[b28] Romero-Lanman E. E., Pavlovic S., Amlani B., Chin Y. & Benezra R. Id1 maintains embryonic stem cell self-renewal by up-regulation of Nanog and repression of Brachyury expression. Stem cells and development 21, 384–393, 10.1089/scd.2011.0428 (2012). 22013995

[b29] Patel D., Morton D. J., Carey J., Havrda M. C. & Chaudhary J. Inhibitor of differentiation 4 (ID4): From development to cancer. Biochimica et biophysica acta 1855, 92–103, 10.1016/j.bbcan.2014.12.002 (2015). 25512197 PMC4312723

[b30] Oatley M. J., Kaucher A. V., Racicot K. E. & Oatley J. M. Inhibitor of DNA binding 4 is expressed selectively by single spermatogonia in the male germline and regulates the self-renewal of spermatogonial stem cells in mice. Biology of reproduction 85, 347–356, 10.1095/biolreprod.111.091330 (2011). 21543770 PMC3142260

[b31] Chan F. *et al.* Functional and molecular features of the Id4+ germline stem cell population in mouse testes. Genes & development 28, 1351–1362, 10.1101/gad.240465.114 (2014). 24939937 PMC4066404

[b32] Suzuki H., Sada A., Yoshida S. & Saga Y. The heterogeneity of spermatogonia is revealed by their topology and expression of marker proteins including the germ cell-specific proteins Nanos2 and Nanos3. Developmental biology 336, 222–231, 10.1016/j.ydbio.2009.10.002 (2009). 19818747

[b33] Zheng K., Wu X., Kaestner K. H. & Wang P. J. The pluripotency factor LIN28 marks undifferentiated spermatogonia in mouse. BMC developmental biology 9, 38, 10.1186/1471-213X-9-38 (2009). 19563657 PMC2719617

[b34] Gassei K. & Orwig K. E. SALL4 expression in gonocytes and spermatogonial clones of postnatal mouse testes. Plos One 8, e53976, 10.1371/journal.pone.0053976 (2013). 23326552 PMC3543410

[b35] Huckins C. The spermatogonial stem cell population in adult rats. I. Their morphology, proliferation and maturation. The Anatomical record 169, 533–557, 10.1002/ar.1091690306 (1971). 5550532

[b36] Oakberg E. F. Spermatogonial stem-cell renewal in the mouse. The Anatomical record 169, 515–531, 10.1002/ar.1091690305 (1971). 5550531

[b37] de Rooij D. G. & Russell L. D. All you wanted to know about spermatogonia but were afraid to ask. Journal of andrology 21, 776–798 (2000). 11105904

[b38] de Rooij D. G. & Griswold M. D. Questions about spermatogonia posed and answered since 2000. Journal of andrology 33, 1085–1095, 10.2164/jandrol.112.016832 (2012). 22879526

[b39] Yoshida S., Nabeshima Y. & Nakagawa T. Stem cell heterogeneity: actual and potential stem cell compartments in mouse spermatogenesis. Annals of the New York Academy of Sciences 1120, 47–58, 10.1196/annals.1411.003 (2007). 17905929

[b40] Griswold M. D. & Oatley J. M. Concise review: Defining characteristics of mammalian spermatogenic stem cells. Stem cells 31, 8–11, 10.1002/stem.1253 (2013). 23074087 PMC5312674

[b41] Soriano P. Generalized lacZ expression with the ROSA26 Cre reporter strain. Nature genetics 21, 70–71, 10.1038/5007 (1999). 9916792

[b42] Barker N. & Clevers H. Lineage tracing in the intestinal epithelium. Current protocols in stem cell biology Chapter 5, Unit5A 4, 10.1002/9780470151808.sc05a04s13 (2010). 20443207

