# Supplementary material for: Id4 Marks Spermatogonial Stem Cells in the Mouse Testis
Source: Sci Rep. 2015 Dec 1;5:17594. doi: 10.1038/srep17594 (PMC4665196; doi:10.1038/srep17594)

Id4 Marks Spermatogonial Stem Cells in the Mouse Testis

Feng Sun[1,*], Qing Xu[1], Danfeng Zhao[1], and Charlie Degui Chen[1,*]

[1] State Key Laboratory of Molecular Biology, Shanghai Key laboratory of Molecular Andrology, Institute of Biochemistry and Cell Biology, Shanghai Institutes for Biological Sciences, Chinese Academy of Sciences, Shanghai 200031, China

[*] Correspondence to: Dr. Feng Sun

Prof. Charlie Degui Chen

Rm.805, Floor 8, Cell Building, 320 Yueyang Road, Shanghai, China

Tel: +86 21 54921148

Fax: +86 21 54921148

E-mail: Feng Sun, fengsun@outlook.com;

Charlie Degui Chen, cdchen@sibcb.ac.cn

Supplemental Figure Legends

Supplemental Figure 1.

a. Representative images of immunostaining for GFRA1 in cross-sections of seminiferous tubules from testes of Id4 knock-in mice.

b. Representative images of immunostaining for Id4 in cross-sections of seminiferous tubules from testes of Id4 knock-in mice. Scale bar, 50 μm.

Supplemental Figure 2. No marking was noted in control mice

a. Whole-mount seminiferous tubule X-gal staining of wild-type mice treated with TM (n = 6). No signal was detected.

b.Whole-mount seminiferous tubule X-gal staining of Id4-CreERT2-tdTomato; ROSA26-flox-stop-lacZ double transgenic mice that were treated with vehicle(n = 5). No signal was detected.

Supplemental Figure 3. Spermatogenesis was not affected in wild-type mice treated with DTx

a. Experimental strategy for DTx treatment.

b. Intact testes from PBS-treated (Ctr) and DTx-treated (DTx) mice. Wild-type mice were treated with PBS or DTx after TM induction. There is no significant difference in testicular morphology between control mice (left column, n = 5) and DTx-treated mice (right column, n = 5). Scale bar, 1 mm.

c. H&E staining of paraffin-embedded sections from the testes of wild-type mice treated with PBS (Ctr, left column) or with DTx (DTx, right column). There is no significant difference between control mice (left column) and DTx-treated mice (right

column). Scale bar, 50 μm.

Supplemental Figure 4. The mean number of $PLZF^+$ cells per tubule cross-section was significantly decreased 8 days after busulfan treatment, compared with that of controls.

a. Immunofluorescence staining for PLZF in cross-sections of seminiferous tubules obtained from the mice 0, 4, and 8 days after busulfan treatment. Scale bar, 50 μm.

b. The mean number of $PLZF^+$ cells per tubule cross-section was significantly decreased after busulfan treatment, compared with that of controls. Three mice were analyzed for each time point. Error bars denote SEM.

Supplemental Figure 1.

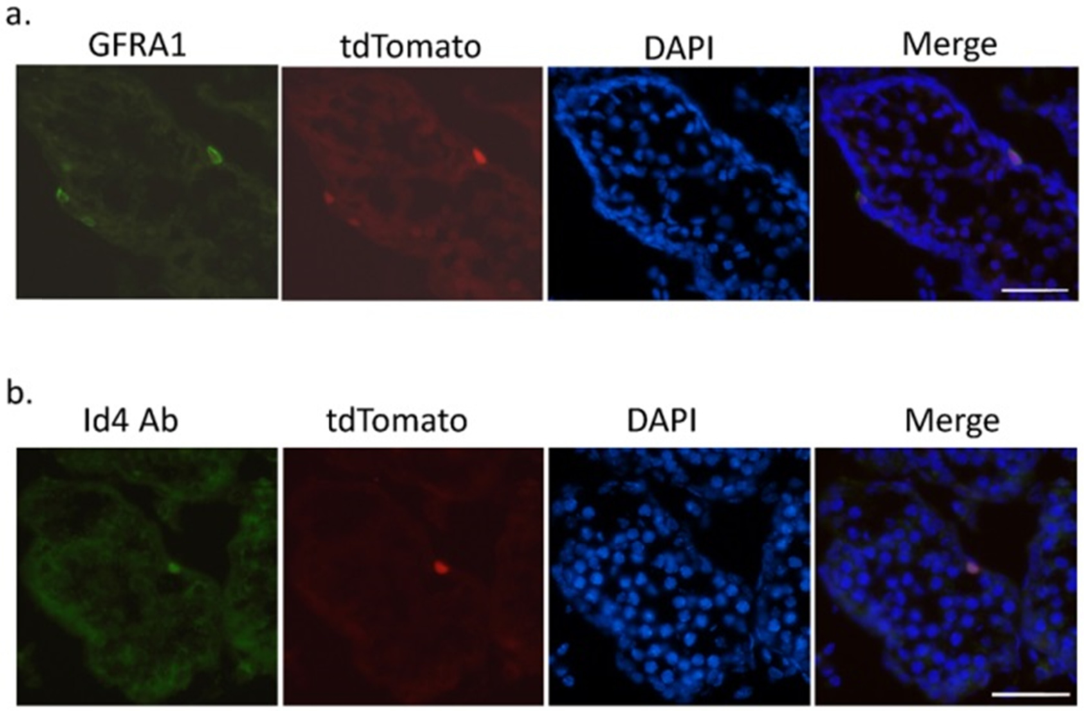

Supplemental Figure 2.

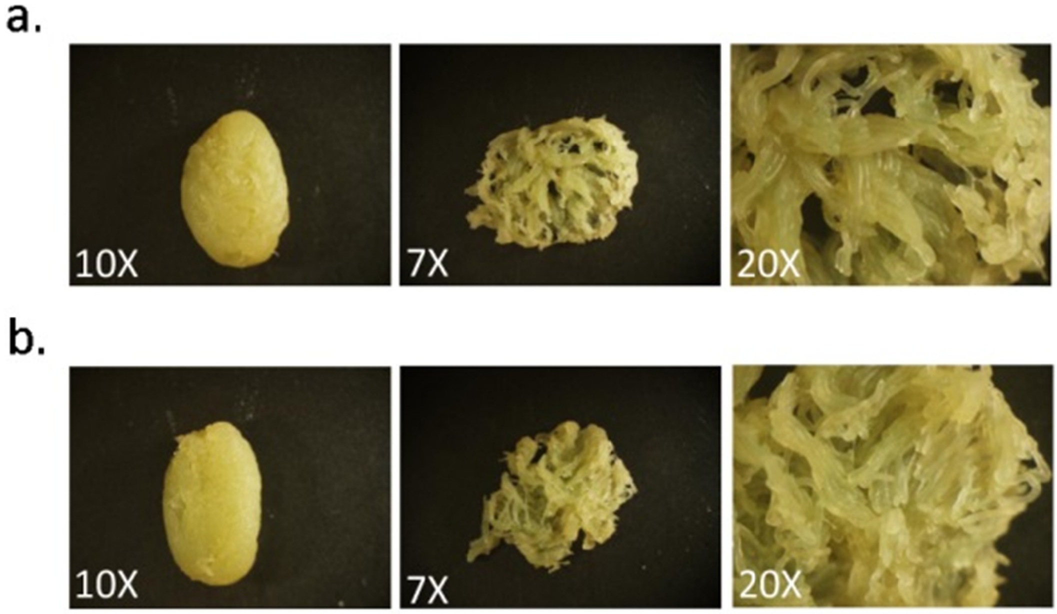

Supplemental Figure 3.

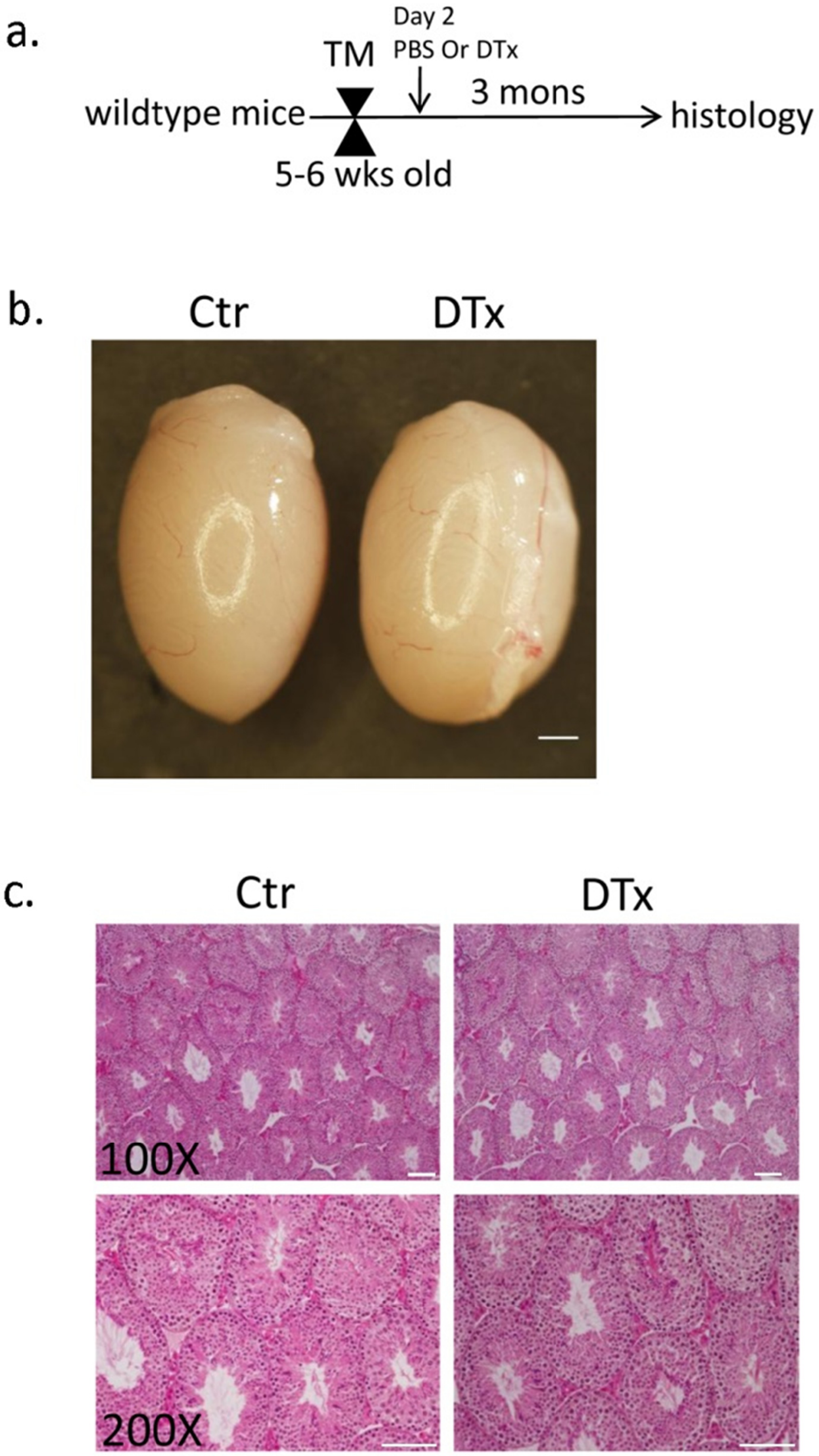

Supplemental Figure 4.

a.

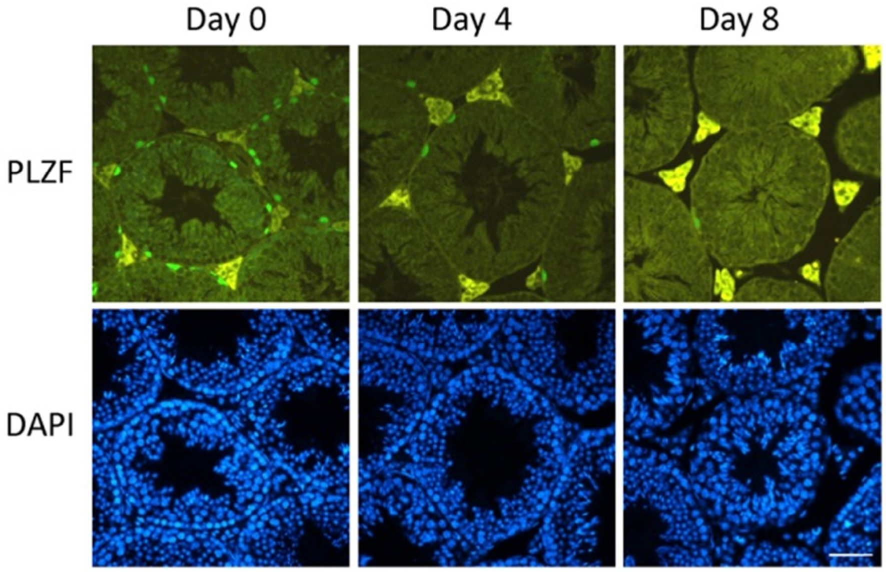

b.

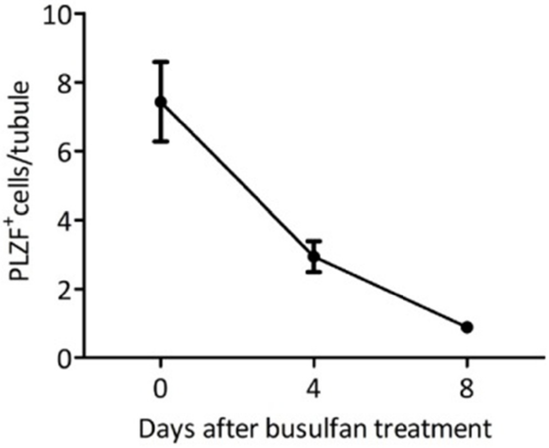

Supplement: Supplementary Information [file srep17594-s1.pdf]
